# Priority-Aware Spectrum Management for QoS Optimization in Vehicular IoT

**DOI:** 10.3390/s25113342

**Published:** 2025-05-26

**Authors:** Adeel Iqbal, Tahir Khurshaid, Yazdan Ahmad Qadri, Ali Nauman, Sung Won Kim

**Affiliations:** 1 School of Computer Science and Engineering, Yeungnam University, Gyeongsan-si 38541, Republic of Korea; adeeliqbal@yu.ac.kr (A.I.); swon@yu.ac.kr (S.W.K.); 2 Department of Electrical Engineering, Yeungnam University, Gyeongsan-si 38541, Republic of Korea; tahir@ynu.ac.kr

**Keywords:** 6G, V-IoT, CTMC, QoS, spectrum utilization

## Abstract

Vehicular Internet of Things (V-IoT) networks, sustained by a high-density deployment of roadside units and sensor-equipped vehicles, are currently at the edge of next-generation intelligent transportation system evolution. However, offering stable, low-latency, and energy-efficient communication in such heterogeneous and delay-prone environments is challenging due to limited spectral resources and diverse quality of service (QoS) requirements. This paper presents a Priority-Aware Spectrum Management (PASM) scheme for IoT-based vehicular networks. This dynamic spectrum access scheme integrates interweave, underlay, and coexistence modes to optimize spectrum utilization, energy efficiency, and throughput while minimizing blocking and interruption probabilities. The algorithm manages resources efficiently and gives proper attention to each device based on its priority, so all IoT devices, from high to low priority, receive continuous and reliable service. A Continuous-Time Markov Chain (CTMC) model is derived to analyze the proposed algorithm for various network loads. Simulation results indicate improved spectral efficiency, throughput, delay, and overall QoS compliance over conventional access methods. These findings establish that the proposed algorithm is a scalable solution for dynamic V-IoT environments.

## 1. Introduction

Vehicular Internet of Things (V-IoT) is an essential component of modern intelligent transportation systems (ITSs), facilitating seamless connectivity between vehicles, roadside units (RSUs), and critical infrastructure [1,2]. With the increasing deployment of autonomous vehicles, vehicle-to-everything (V2X) communication, and smart mobility solutions, ensuring optimal quality of service (QoS) in vehicular networks has become a major challenge [3]. The highly dynamic nature of V-IoT, characterized by high mobility, fluctuating traffic loads, and limited spectrum availability, demands an adaptive and intelligent spectrum management framework to optimize resource allocation while maintaining network reliability and efficiency.

Spectrum accessibility mechanisms are often static and are not adapted to the heterogeneous and dynamic nature of V-IoT communications [4]. Innovative spectrum access methodologies such as interweave and underlay are introduced to maximize spectrum utilization [5]. However, these methodologies do not sufficiently understand pertinent critical QoS metrics such as interruption probability, blocking probability, and energy efficiency. Blocking probability quantifies the likelihood that a communication request is blocked due to spectrum unavailability. In contrast, interruption probability represents the risk of an ongoing session being interrupted due to preemption or interference. Energy efficiency is essential in sustainable V-IoT communication, as high power consumption can deplete vehicular battery life and introduce uncertainties. Besides these, spectrum utilization and throughput are measures of performance gauging the efficiency of spectrum allocation to optimize the use of available resources. Extended data delivery time (EDDT) is an appropriate metric for evaluating V-IoT network performance as it considers delays due to spectrum handoffs, waiting schemes, and preemptive resource allocation. Furthermore, steady-state probability calculation can help understand the long-term behavior as it computes the probability of the network being in a specific state under varying traffic conditions.

The existing literature is primarily focused on throughput optimization and latency reduction, and it tends to overlook the overall impact of spectrum congestion, service disruption, and energy consumption on network performance [6,7,8]. Overcoming these challenges facilitates smooth and efficient vehicular communication, particularly in mission-critical applications such as emergency vehicle communication, intelligent traffic management, and critical missions. This paper introduces a Priority-Aware Spectrum Management (PASM) for IoT networks to address these limitations. This novel spectrum access framework aims to maximize spectrum allocation for multi-priority V-IoT users. The solution addresses the limitations of the solution presented in [8] and builds on its model that dynamically switches between interweave, underlay, and coexistence modes to further balance spectrum efficiency, reduce blocking probability, minimize service interruption, and improve energy efficiency while maintaining high throughput and QoS compliance. To evaluate the effectiveness of the proposed solution, we consider the Continuous-Time Markov Chain (CTMC) model presented in [8] that effectively models the behavior of multi-class V-IoT users accessing the spectrum in various network scenarios. From the CTMC model, we utilize key performance metrics and provide an extensive comparison with traditional spectrum access solutions. Extensive simulation results demonstrate that the proposed solution significantly enhances overall spectrum utilization, improves communication reliability, and conserves energy. The proposed framework is an extensible and robust solution for next-generation V-IoT networks that offers a future research platform for adaptive spectrum management.

In realistic vehicular scenarios, not all vehicles are equipped with communication capabilities. This leads to a Mixed Connected and Connectionless Vehicle (MCCV) environment, where connected vehicles with On-Board Units (OBUs) and non-connected vehicles without OBUs coexist on the same road infrastructure. Recent work in [9] demonstrates that communication asymmetry in MCCV settings complicates coordinated maneuvers, such as overtaking, and introduces challenges in cooperative decision-making. While the proposed PASM framework targets spectrum access among connected vehicles, the presence of non-connected vehicles introduces uncertainty in channel demand and dynamic scheduling. In this study, non-connected vehicles are treated as passive elements that do not interact with the spectrum manager and are excluded from resource allocation processes. Future extensions of this work will address the impact of non-connected traffic by incorporating dynamic spectrum modeling that reflects indirect effects such as interference variability, mobility unpredictability, and priority adjustments for safety-critical broadcasts.

The remainder of this paper is structured as follows. Section 2 provides a detailed overview of existing spectrum access techniques. Section 3 details the proposed system architecture and spectrum access framework, the proposed algorithm, and the CTMC-based analytical model for evaluating spectrum access performance. Section 4 discusses the simulation results and evaluates the performance of the proposed solution against existing approaches. Finally, Section 5 concludes this study with key findings and future research directions.

## 2. Related Works

The growing demand for efficient and reliable spectrum access in V-IoT networks has led to extensive research efforts to address the challenges in dynamic spectrum management [10]. Traditional static spectrum allocation methods fail to adapt to the dynamic nature of vehicular networks, leading to inefficient resource utilization and increased service interruptions. To address these challenges, several studies have proposed hybrid spectrum access techniques, adaptive resource allocation mechanisms, and QoS-aware communication models [11,12]. However, the applicability of these solutions in high-mobility vehicular environments with diverse priority levels remains an open problem.

The existing spectrum access techniques for vehicular networks can be broadly categorized into three primary approaches: fixed allocation, interweave access, and underlay sharing. Each class offers trade-offs in complexity, spectral efficiency, and latency guarantees.

Fixed spectrum allocation methods assign preallocated frequency bands for vehicular communication, typically regulated by spectrum authorities. While this approach simplifies access control, it leads to spectrum under-utilization in low-traffic conditions and severe congestion in high-density environments, particularly during peak hours or urban bottlenecks [13]. As vehicular traffic patterns vary rapidly with time and location, static allocation schemes fall short of delivering consistent performance.

Interweave spectrum access allows V-IoT users to opportunistically exploit idle frequency bands when no active primary users are present. This method offers high throughput when spectrum holes are available and has been widely studied in the cognitive radio literature [14]. Nonetheless, it suffers from frequent spectrum handoffs due to primary user reappearance, which leads to increased latency and degraded link stability. Furthermore, it requires highly accurate spectrum sensing, which may not be feasible in high-speed vehicular scenarios where signal detection windows are limited.

Underlay spectrum access enables simultaneous sharing of the spectrum between primary and secondary users by imposing strict interference thresholds. In such schemes, V-IoT users transmit at reduced power to avoid disrupting licensed users [15]. While this approach reduces blocking probability and improves overall channel utilization, it generally results in lower energy efficiency due to power limitations and requires robust interference management techniques to maintain system integrity.

To overcome the individual limitations of interweave and underlay methods, researchers have explored hybrid spectrum access techniques that dynamically switch between the two modes based on real-time traffic conditions, channel availability, or QoS requirements. These approaches attempt to combine the strengths of opportunistic access and controlled coexistence.

Authors in [16] proposed a Continuous-Time Markov Chain (CTMC)-based hybrid access scheme to enhance spectrum utilization in vehicular networks. Their model effectively captured the probabilistic behavior of primary and secondary users; however, it lacked waiting mechanisms for low-priority users, resulting in high blocking probabilities and service denials during congestion. Similarly, the authors in [17] analyzed success probability and age-of-information metrics in hybrid access environments. While their work provides insights into information freshness, it does not consider energy consumption or spectrum efficiency, both of which are crucial in power-constrained V-IoT devices.

To address transmission delays, the authors in [18] introduced a reservation-based medium access control protocol tailored for multi-priority vehicular networks. Although this reduces queuing delays, it inadvertently increases the number of spectrum handoffs, thereby raising the risk of service interruptions. The authors in [10] integrated blockchain into spectrum management to provide decentralized access control and enhanced security. However, their work overlooks energy-aware communication and does not account for rapid changes in vehicular topology, which can significantly affect access performance.

The authors in [19] conducted a transient analysis of multiple spectrum access mechanisms, including hybrid access. Their work provides valuable insights into short-term system behavior but is primarily limited to WiFi environments and does not include metrics like interruption probability or energy efficiency that are critical for long-lived V-IoT sessions.

The authors in [20] focused on optimizing 5G spectrum allocation for agricultural Internet of Things (IoT) applications. Their study compared three resource management models to address call blocking and handover issues, offering valuable techniques for rural deployments. However, the absence of throughput and latency analysis limits its application to real-time vehicular networks where low-latency data delivery is paramount.

Several CTMC-based models integrate interweave and underlay modes for dynamic access, especially in cognitive radio networks (CRNs). The authors in [14] provided a comparative study of interweave and underlay performance but did not consider the presence of heterogeneous priority classes or real-time vehicular traffic models. Most CRN-focused works fail to address the service requirements of primary users in multi-tier vehicular networks, which include safety-critical and infotainment applications.

Recent studies have increasingly applied machine learning to V-IoT spectrum access. For instance, the authors in [21] proposed a federated deep reinforcement learning (FDRL) framework for dynamic spectrum access in IoT networks. Their model enables distributed learning across multiple nodes without sharing raw data, preserving privacy and improving learning efficiency. The FDRL agent optimizes channel access policies in a decentralized manner, showing improved spectrum efficiency under non-stationary environments. However, the model assumes consistent federated training and computational capacity in all devices, which may not always be feasible in highly mobile or resource-constrained vehicular environments. The authors in [22] proposed a hierarchical adaptive federated reinforcement learning (HAFRL) framework for resource allocation and task scheduling in hierarchical IoT networks. Their approach integrates local learning at edge nodes with global coordination at cloud servers, adapting to dynamic network conditions while preserving data privacy. Although highly relevant for distributed vehicular networks, the study does not incorporate priority-aware spectrum access or consider the queuing mechanisms required for multi-class V-IoT traffic scenarios.

The authors in [23] introduced a multi-agent reinforcement learning approach using a Dueling Double Deep Q-Network (D3QN) for efficient resource allocation in vehicular networks. Their framework allows distributed decision-making among multiple agents, optimizing network throughput while considering environmental dynamics. Although the method effectively manages radio resources in complex vehicular environments, it does not model heterogeneous traffic priorities or incorporate interruption-aware access mechanisms required for fairness in V-IoT scenarios.

Our earlier work in [8] introduced an adaptive multi-mode spectrum access model and a hybrid CTMC scheme for V-IoT environments. It achieved significant improvements in throughput and spectrum efficiency while reducing access delay for low-priority users. Nevertheless, that study did not evaluate key reliability metrics, such as interruption and blocking probability, or account for energy efficiency in high-mobility settings. Furthermore, it lacked provisions for waiting mechanisms or handoff handling strategies essential for fairness in access. The authors in [24] proposed a blockchain-based two-stage secure spectrum auction framework that integrates intelligent sensing and sharing mechanisms. Their design leverages auction theory and distributed ledgers to enhance trust, transparency, and security in dynamic spectrum markets. While highly effective in static industrial IoT contexts, the proposed approach does not address real-time vehicular mobility, multi-priority queuing, or adaptive coexistence, which are crucial for V-IoT networks.

Recent efforts, such as [8,25], have attempted to broaden evaluation metrics to include handoff probability, service capacity, and spectrum utilization. However, these works still fall short of delivering a unified model that addresses all major QoS indicators for multi-priority V-IoT systems.

Table 1 tabulates the state-of-the-art spectrum access models, highlighting their key contributions and critical limitations, which motivate the need for a more holistic and scalable framework.

## 3. System Model

The proposed framework addresses the challenges of dynamic spectrum management in V-IoT networks by ensuring efficient spectrum use and QoS differentiation across different priority classes. Vehicles in the heterogeneous environment, which are equipped with IoT sensors and communication modules, exchange critical information with roadside units, satellite relays, and fifth-generation New Radio (5G-NR) base stations (BSs) under regulatory spectrum access laws. Devices are assigned three priority classes: top-priority devices (TID) for safety-critical services, medium-priority devices (MID) for traffic management and infotainment, and low-priority devices (LID) for non-urgent telemetry. Spectrum access is dynamically managed in interweave, underlay, and coexistence modes.

**Figure 1:**
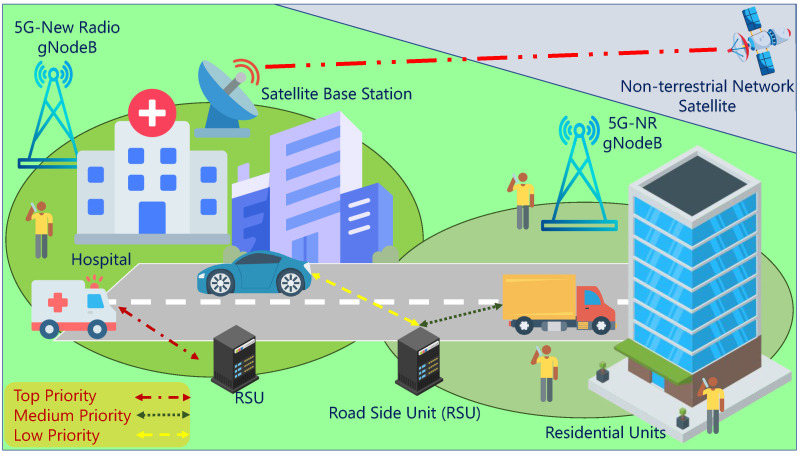
System model: V-IoT system in a smart city environment.

The tri-modal scheme ensures that high-priority traffic has negligible delay, medium-priority traffic has guaranteed throughput, and low-priority traffic has a waiting mechanism to prevent outright session drops. The communication environment also includes corporate fleets, public transit systems, emergency services, medical transport, and pedestrian phones to offer an overlay of urban and vehicular sensor networks. The model makes integrating satellite, cellular, and roadside connectivity easy to support the robust, low-latency communication needed for intelligent transportation systems in smart city applications. Figure 1 shows the architecture of this integrated V-IoT environment in depth.

The proposed framework facilitates efficient and adaptive spectrum utilization by dynamically reallocating resources based on both the network environment and the priority class of users. It supports three distinct spectrum access modes—interweave, underlay, and coexistence—each tailored to ensure service quality across heterogeneous vehicular IoT traffic classes.

In the interweave mode, V-IoT nodes are permitted to access the spectrum only when it is determined to be idle, i.e., when no higher-priority users (such as TID or MID devices) are occupying the channel. This mode ensures interference-free communication and is suitable for delay-tolerant or non-critical data transmissions. The maximum achievable data rate for a user γ operating in interweave mode is given by

(1) $$d_{i}=W\log_{2}\left(1+\frac{p_{\gamma }g_{\gamma }}{N}\right)$$

In this expression, $d_{i}$ denotes the data rate in bits per second, *W* is the available channel bandwidth in hertz, $p_{\gamma }$ represents the transmission power of user γ, $g_{\gamma }$ is the channel gain between the transmitting user and its receiver, and *N* is the power of additive white Gaussian noise (AWGN) affecting the channel. This formulation assumes that no interference from other users is present, consistent with the interweave mode’s opportunistic nature.

In the underlay mode, the system permits concurrent transmissions from lower-priority users (such as MID and LID) alongside high-priority TID users, under the constraint that the interference caused by the lower-priority users remains below a predefined threshold. This mode enables higher spectrum utilization but requires precise power control. The maximum data rate achievable for a user γ under underlay conditions is given by

(2) $$d_{u}=W\log_{2}\left(1+\frac{p_{\gamma }g_{\gamma }}{N+p_{t}g_{t,\gamma }}\right)$$

Here, $d_{u}$ is the achievable data rate in underlay mode, $p_{t}$ is the transmission power of the coexisting top-priority TID user, and $g_{t,\gamma }$ is the cross-channel gain from the TID user to the receiver of user γ. The term $N+p_{t}g_{t,\gamma }$ in the denominator represents the combined impact of noise and interference experienced by the lower-priority user due to the TID transmission. The reduced signal-to-interference-plus-noise ratio (SINR) in this scenario directly affects achievable throughput.

In the coexistence mode, the framework introduces a hybrid mechanism that allows flexible transitions between interweave and underlay access, with an added waiting mechanism for LID users. Rather than being dropped immediately when the spectrum is occupied, LID users are placed in a temporary buffer (waiting queue) and granted access once resources are freed. If the wait exceeds a threshold, spectrum handoff to an alternative band is initiated. This mechanism ensures fairness by minimizing service disruption for low-priority users. A Preemptive Resume Priority (PRP) queuing discipline is employed, allowing TID and MID users to preempt ongoing LID sessions, with LID transmissions resuming once the higher-priority session ends.

Spectrum availability is determined through channel sensing, modeled by the binary hypothesis test:

(3) $$x\left(t\right)=\left\{\begin{array}{cc} n(t), & H_{0}\;\left(\mathrm{channel}\;\mathrm{is}\;\mathrm{idle}\right)\\ c(t)+n(t), & H_{1}\;\left(\mathrm{channel}\;\mathrm{is}\;\mathrm{active}\right) \end{array}\right.$$

In this model, x(t) is the received signal, n(t) represents the background noise modeled as AWGN, and c(t) is the signal transmitted by a high-priority user. Hypothesis $H_{0}$ denotes an idle channel, while $H_{1}$ indicates an active channel. For analytical tractability, we assume perfect sensing in this study, meaning the system can accurately detect whether the channel is idle or occupied without false alarms or missed detections. The impact of this simplifying assumption and its potential relaxation through adaptive sensing techniques is discussed in later sections.

The proposed framework has several advantages over traditional spectrum access frameworks. It provides priority-aware access control, maximum spectrum utilization, and seamless service continuity to numerous V-IoT applications. The framework optimizes the trade-offs between blocking probability, energy efficiency, and interruption probability and is, hence, a promising solution for future vehicular networks.

### 3.1. Proposed Methodology

The proposed priority-aware spectrum management for V-IoT employs a hierarchical approach to spectrum allocation, where access to the spectrum is dynamically determined under varying conditions. It initially considers opportunistic access, i.e, interweave mode. Without an idle channel, the model provides simultaneous transmission subject to interference limitations, operating in the underlay mode. If opportunistic access or simultaneous transmission is impossible, the system adopts a temporary waiting approach instead of dropping connections to ensure continuity through the coexistence mode. This dynamic approach enhances spectrum utilization without compromising QoS for V-IoT applications.

### 3.2. CTMC Modeling of Spectrum Access Schemes

In this section, we present the CTMC modeling of two existing schemes, namely, interweave spectrum access [14,16] and hybrid interweave underlay spectrum access [8,16,17,26]. Finally, our proposed CMTC model is presented.

#### 3.2.1. Interweave Spectrum Access

**Figure 2:**
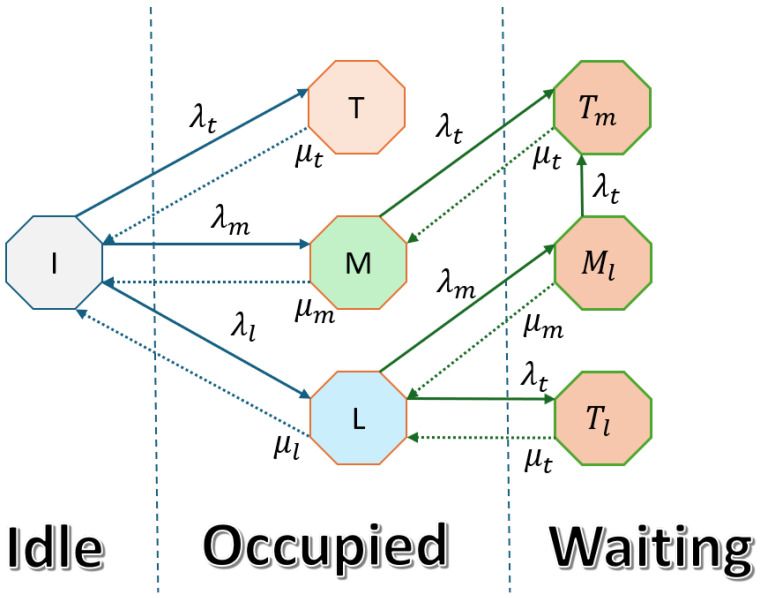
Interweave spectrum access mechanism CTMC.

This section considers a vehicular network with nodes belonging to TID, MID, and LID categories. The state transition diagram for interweave spectrum access containing idle, occupied, and waiting states is shown in Figure 2. The state *I* represents that the system is idle, while T,M,L represent that the system is occupied by TID, MID, and LID users, respectively. The arrival rates of TID, MID, and LID are $\lambda_{t},\lambda_{m}$, and $\lambda_{l}$; similarly, the departure rates of TID, MID, and LID are $\mu_{t},\mu_{m}$, and $\mu_{l}$. The TID holds the highest priority, which means it will immediately occupy the spectrum without considering its state, and MID and LID users will go into a waiting state on the arrival of the TID. $T_{m}$ represents the MID user moving to the waiting state on the arrival of the TID, while $M_{l}$ represents the transition of the LID user to the waiting state on the arrival of an MID user. If the system is in state $M,M_{l},L$ and there is an arrival of a TID user with $\lambda_{t}$, the system will move from the $M,M_{l}$ state to the $T_{m}$ state and from the *L* to $T_{l}$ state. In the case of $M_{l}$, the LID user will be dropped from the system because only one user can stay waiting. The system is in state *L*, and the MID will arrive with $\lambda_{m}$, so the system will move into the $M_{l}$ state. TID users can preempt MID and LID, MID can preempt LID, and only one user can wait. If two users are in the waiting state, the lower-priority user will be dropped from the system.

The state-space vector of this system is $S=\left\{I,T,M,L,T_{m},T_{l},M_{l}\right\}$. We denote this CTMC as “Interweave”, and the transition rate matrix *Q* is given in Equation (4).

(4) Q=    I    T  M     L   Tm Tl  Ml  ITMLTmTlMl−(λt+λm+λl)λtλmλl000μt−μt00000μm0−(μm+λt)0λt00μl00−(μl+λt+λm)0λtλm00μt0−μt00000μt0−μt0000μmλc0−(μm+λc)

Equation (5) provides a combination of flow balance equations for interweave spectrum access.

(5) $$\left.\begin{array}{c} \pi_{i}\left(\lambda_{t}+\lambda_{l}+\lambda_{m}\right)=\pi_{t}\mu_{t}+\pi_{m}\mu_{m}+\pi_{l}\mu_{l},\\ \pi_{t}\mu_{t}=\pi_{i}\lambda_{t},\\ \pi_{m}\left(\lambda_{t}+\mu_{m}\right)=\pi_{i}\lambda_{m}+\pi_{t_{m}}\mu_{t},\\ \pi_{l}\left(\lambda_{t}+\mu_{l}+\lambda_{m}\right)=\pi_{i}\lambda_{l}+\pi_{t_{l}}\mu_{t}+\pi_{m_{l}}\mu_{m},\\ \pi_{t_{m}}\mu_{t}=\pi_{m}\lambda_{t}+\pi_{m_{l}}\lambda_{t},\\ \pi_{t_{l}}\mu_{t}=\pi_{l}\lambda_{t},\\ \pi_{m_{l}}\left(\lambda_{t}+\mu_{m}\right)=\pi_{l}\lambda_{m}, \end{array}\right.$$

Equation (6) provides the normalization condition:

(6) $$\sum_{\beta \in S}\pi_{\beta }=1,$$

By solving the set of derived Equations (5) and (6), we obtain steady-state probabilities $\pi_{i},\pi_{t},\pi_{m},\pi_{l},\pi_{t_{m}},\pi_{t_{l}},\pi_{m_{l}}$, which are given by

(7) $$\left.\begin{array}{c} \pi_{i}=\frac{\mu_{m}\left(\lambda_{m}\lambda_{t}+\left(\lambda_{t}+\mu_{m}\right)\mu_{l}\right)\mu_{t}}{\left(\lambda_{m}+\mu_{m}\right)\left(\lambda_{m}\lambda_{t}+\left(\lambda_{t}+\mu_{m}\right)\left(\lambda_{l}+\mu_{l}\right)\right)\left(\lambda_{t}+\mu_{t}\right)},\\ \pi_{t}=\frac{\lambda_{t}\mu_{m}\left(\lambda_{m}\lambda_{t}+\left(\lambda_{t}+\mu_{m}\right)\mu_{l}\right)}{\left(\lambda_{m}+\mu_{m}\right)\left(\lambda_{m}\lambda_{t}+\left(\lambda_{t}+\mu_{m}\right)\left(\lambda_{l}+\mu_{l}\right)\right)\left(\lambda_{t}+\mu_{t}\right)},\\ \pi_{m}=\frac{\lambda_{m}\left(\left(\lambda_{m}+\lambda_{l}\right)\lambda_{t}+\left(\lambda_{t}+\mu_{m}\right)\mu_{l}\right)\mu_{t}}{\left(\lambda_{m}+\mu_{m}\right)\left(\lambda_{m}\lambda_{t}+\left(\lambda_{t}+\mu_{m}\right)\left(\lambda_{l}+\mu_{l}\right)\right)\left(\lambda_{t}+\mu_{t}\right)},\\ \pi_{d_{l}}=\frac{\lambda_{l}\mu_{m}\left(\lambda_{t}+\mu_{m}\right)\mu_{t}}{\left(\lambda_{m}+\mu_{m}\right)\left(\lambda_{m}\lambda_{t}+\left(\lambda_{t}+\mu_{m}\right)\left(\lambda_{l}+\mu_{l}\right)\right)\left(\lambda_{t}+\mu_{t}\right)},\\ \pi_{t_{m}}=\frac{\lambda_{m}\lambda_{t}}{\left(\lambda_{m}+\mu_{m}\right)\left(\lambda_{t}+\mu_{t}\right)},\\ \pi_{t_{l}}=\frac{\lambda_{l}\lambda_{t}\mu_{h}\left(\lambda_{t}+\mu_{m}\right)}{\left(\lambda_{m}+\mu_{m}\right)\left(\lambda_{m}\lambda_{t}+\left(\lambda_{t}+\mu_{m}\right)\left(\lambda_{l}+\mu_{l}\right)\right)\left(\lambda_{t}+\mu_{t}\right)},\\ \pi_{m_{l}}=\frac{\lambda_{m}\lambda_{l}\mu_{m}\mu_{t}}{\left(\lambda_{m}+\mu_{m}\right)\left(\lambda_{m}\lambda_{t}+\left(\lambda_{t}+\mu_{m}\right)\left(\lambda_{l}+\mu_{l}\right)\right)\left(\lambda_{t}+\mu_{t}\right)}. \end{array}\right.$$

#### 3.2.2. Hybrid Interweave Underlay Spectrum Access

**Figure 3:**
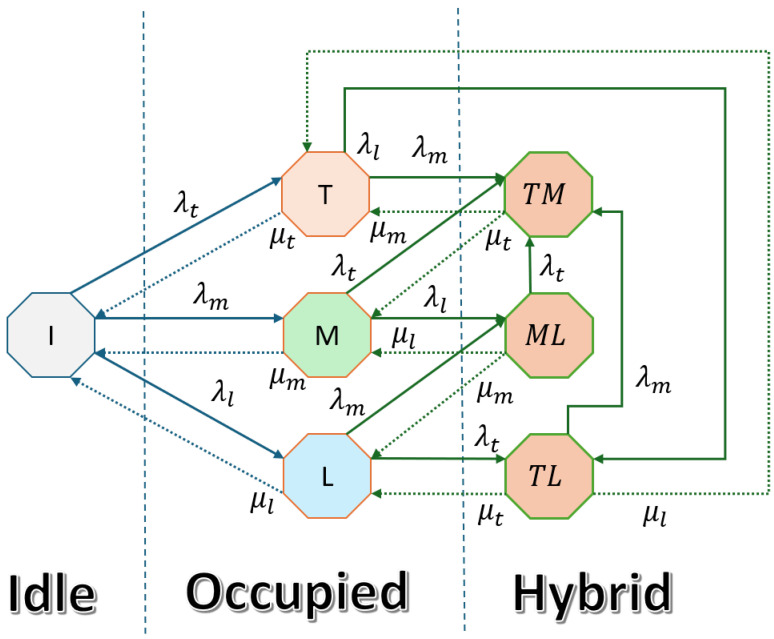
Hybrid interweave underlay spectrum access CTMC.

The hybrid interweave underlay spectrum access mechanism [8,16], also called hybrid spectrum access, allows two devices to simultaneously utilize the spectrum without causing significant interference. This is achieved by strictly adhering to interference avoidance mechanisms and maintaining power levels below a predefined threshold. The lower-priority device reduces its transmission power, potentially compromising its data rate while ensuring the communication session remains active. Figure 3 presents the state transition diagram of the hybrid interweave underlay spectrum access mechanism.

(8) Q=      I      T      M      L    TM     TL     ML     ITMLTMTLML−(λt+λm+λl)λtλmλl000μt−(μt+λm+λl)00λmλl0μm0−(μm+λt+λl)0λt0λlμl00−(μl+λt+λm)0λtλm0μmμt0−(μt+μm)000μl0μtλm−(μt+μl+λm)000μlμmλt0−(μm+μl+λc)

The state transition model for the hybrid interweave underlay spectrum access can be described using the following balance equations:

(9) $$\begin{array}{c} \pi_{i}\left(\lambda_{t}+\lambda_{l}+\lambda_{m}\right)=\pi_{t}\mu_{t}+\pi_{m}\mu_{m}+\pi_{l}\mu_{l},\\ \pi_{t}\left(\mu_{t}+\lambda_{l}+\lambda_{m}\right)=\pi_{i}\lambda_{t}+\pi_{tl}\mu_{l}+\pi_{tm}\mu_{m},\\ \pi_{m}\left(\lambda_{t}+\mu_{m}+\lambda_{l}\right)=\pi_{i}\lambda_{m}+\pi_{tm}\mu_{t}+\pi_{ml}\mu_{l},\\ \pi_{l}\left(\lambda_{t}+\mu_{l}+\lambda_{m}\right)=\pi_{i}\lambda_{l}+\pi_{tl}\mu_{t}+\pi_{ml}\mu_{m},\\ \pi_{tm}\left(\mu_{t}+\mu_{m}\right)=\pi_{m}\lambda_{t}+\pi_{t}\lambda_{m}+\pi_{tl}\lambda_{m}+\pi_{ml}\lambda_{t},\\ \pi_{tl}\left(\mu_{t}+\mu_{l}+\lambda_{m}\right)=\pi_{l}\lambda_{t}+\pi_{t}\lambda_{l},\\ \pi_{ml}\left(\lambda_{t}+\mu_{m}+\mu_{l}\right)=\pi_{l}\lambda_{m}+\pi_{m}\lambda_{l}. \end{array}$$

The steady-state probabilities for each state $\pi_{\beta }$(β∈S) can be determined by solving the system of linear equations given in (9) along with the normalization condition:

(10) $$\sum_{\beta \in S}\pi_{\beta }=1.$$

The presence of a TID user in operation is obtained by summing over states where TID is active, i.e., $\pi_{t}$, $\pi_{tm}$, and $\pi_{tl}$. Similarly, when MID accesses the spectrum in interweave mode, the steady-state probability is given by $\pi_{m}+\pi_{ml}$. LID only operates in interweave mode in state $\pi_{l}$.

#### 3.2.3. Proposed Spectrum Access Mechanism

Only two higher-priority-category devices can access the system simultaneously in both interweave and hybrid spectrum access. Whenever all three categories of devices, TID, MID, and LID, are present, LID is dropped, and only TID and MID are accommodated in the system. To overcome this limitation, we proposed a solution that enables an advanced spectrum access technique. The overall process remains the same as in the previous models but with the following enhancements.

When the system is in the *T* and *M* states and an LID user arrives with rate $\lambda_{l}$, the system transitions to the $TM_{l}$ state, where TID operates at a full rate without any interruption and MID operates in underlay mode with a reduced rate. At the same time, LID enters a waiting state, waiting for TID or MID to complete its session and vacate the spectrum. Similarly, if the system is at the TL state and an MID arrives with rate $\lambda_{m}$, the system transitions to the $TM_{l}$ state under the same conditions. Likewise, when the system is at the ML state and a TID arrives with rate $\lambda_{t}$, it moves to the $TM_{l}$ state following the same conditions.

**Figure 4:**
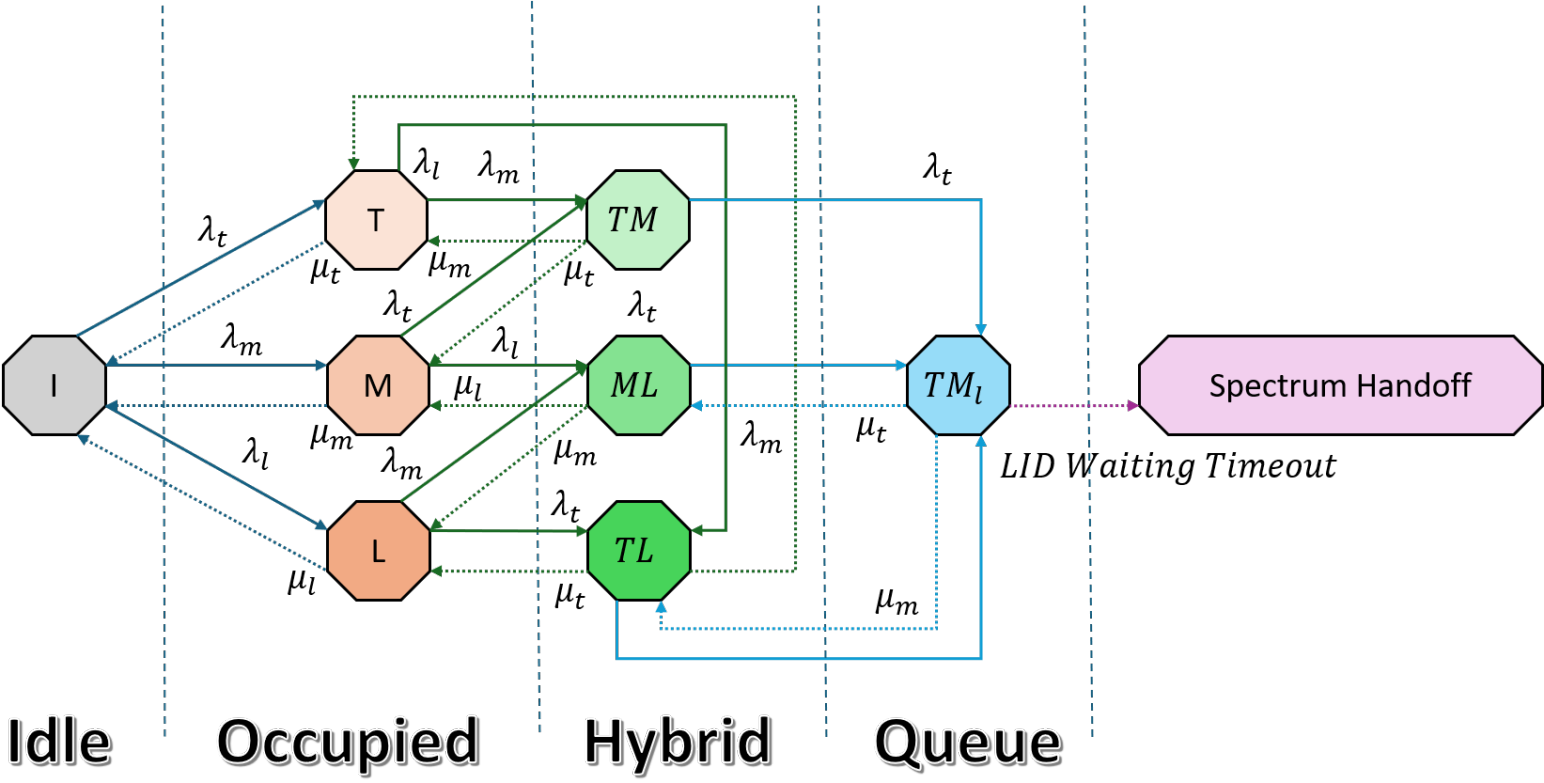
Proposed spectrum access mechanism CTMC.

In the $TM_{l}$ state, TID continues to use the spectrum at the interweave rate, MID operates in underlay mode, and LID remains in a waiting state until its waiting time exceeds a predefined threshold, after which it undergoes spectrum handoff. Figure 4 represents the state transition diagram of the proposed solution, which follows the Algorithm 1.

The transition probability matrix is formulated as

(11) Q=      I      T      M      L      TM     TL     ML    TMl    ITMLTMTLMLTMl−(λt+λm+λl)λtλmλl0000μt−(μt+λm+λl)00λmλl00μm0−(μm+λc+λl)0λc0λl0μl00−(μl+λc+λm)0λcλm00μmμt0−(μt+μm+λl)00λl0μl0μt0−(μt+μl+λm)0λm00μlμm00−(μm+μl+λc)λc00000μmμt−(μt+μm)

(12) $$\begin{array}{c} \pi_{i}\left(\lambda_{t}+\lambda_{l}+\lambda_{m}\right)=\pi_{t}\mu_{t}+\pi_{m}\mu_{m}+\pi_{l}\mu_{l},\\ \pi_{t}\left(\mu_{t}+\lambda_{l}+\lambda_{m}\right)=\pi_{i}\lambda_{t}+\pi_{cl}\mu_{l}+\pi_{tm}\mu_{m},\\ \pi_{m}\left(\lambda_{t}+\mu_{m}+\lambda_{l}\right)=\pi_{i}\lambda_{m}+\pi_{tm}\mu_{t}+\pi_{ml}\mu_{l},\\ \pi_{l}\left(\lambda_{t}+\mu_{l}+\lambda_{m}\right)=\pi_{i}\lambda_{l}+\pi_{tl}\mu_{t}+\pi_{ml}\mu_{m},\\ \pi_{tm}\left(\mu_{t}+\mu_{m}+\lambda_{l}\right)=\pi_{t}\lambda_{m}+\pi_{m}\lambda_{t}+\pi_{{tm}_{l}}\lambda_{l},\\ \pi_{tl}\left(\mu_{t}+\mu_{l}+\lambda_{m}\right)=\pi_{l}\lambda_{t}+\pi_{t}\lambda_{l}+\pi_{{tm}_{l}}\mu_{m},\\ \pi_{ml}\left(\lambda_{t}+\mu_{m}+\mu_{l}\right)=\pi_{l}\lambda_{m}+\pi_{m}\lambda_{l},\\ \pi_{{tm}_{l}}\left(\mu_{t}+\mu_{m}\right)=\pi_{tl}\lambda_{m}+\pi_{tm}\lambda_{l}+\pi_{ml}\lambda_{t}, \end{array}$$

**Algorithm 1** Priority-Aware Spectrum Management (PASM) algorithm**Input:** Channel state *S* (Idle/Busy), IoT device category ∈{TID,MID,LID}, access modes {I: Interweave, U: Underlay, C: Coexistence} **Output:** Optimized spectrum allocation with minimal blocking/interruption **Initialization:** All devices sense the channel to determine availability *S* 1: **if** 
S==Idle 
**then** 2:      Allocate spectrum using Interweave mode 3:      Assign channel to the requesting user based on priority TID arrives 4: **else if** *TID* arrives **then** 5:      **if** S==Idle **then** 6:          Grant immediate access to TID (preempts none) 7:      **else** 8:          Preempt any ongoing MID or LID transmissions 9:          Reassign channel to TID (highest priority) 10:      **end if** 11: **else if** MID arrives **then** 12:      **if** channel occupied by LID **then** 13:          MID preempts LID 14:          LID enters coexistence queue with waiting timer $t_{\mathrm{wait}}$ 15:      **else** 16:          Grant access to MID in Interweave or Underlay mode 17:      **end if** 18: **else if** LID arrives **then** 19:      **if** channel is available **then** 20:          Grant access using Interweave or Underlay mode 21:      **else** 22:          LID enters coexistence mode and starts $t_{\mathrm{wait}}$ 23:          **while** channel is still occupied and $t<t_{\mathrm{wait}}$ **do** 24:               LID remains in queue 25:          **end while** 26:          **if** channel becomes free before timeout **then** 27:               LID resumes transmission 28:          **else** 29:               Perform spectrum handoff to another band 30:          **end if** 31:      **end if** 32: **end if** 33: End session when transmission is complete


The steady-state probabilities for each state $\pi_{\beta }$(β∈S) can be obtained by solving the set of linear equations in (12) along with the normalization equation.

The steady-state probability $\pi_{t}$ is obtained by summing the states where TID is active, which includes $\pi_{T}$, $\pi_{tm}$, $\pi_{tl}$, and $\pi_{tm_{l}}$. Similarly, when MID accesses the spectrum in interweave mode, its steady-state probability is represented by $\pi_{m}$ and $\pi_{ml}$. On the other hand, LID only accesses the system in interweave mode, which is represented by $\pi_{l}$, giving us the steady-state probability $\pi_{l}$.

#### 3.2.4. Comparison of Spectrum Access Mechanisms

To validate the CTMC models and quantify the benefits of the proposed framework, we compare key steady-state probabilities under varying traffic loads using percentage differences. The percentage difference between two values $V_{1}$ and $V_{2}$ is defined as

(13) $$\mathrm{Percentage}\;\mathrm{Difference}=\frac{\left|V_{1}-V_{2}\right|}{\left(\frac{V_{1}+V_{2}}{2}\right)}\times 100.$$

**Figure 5:**
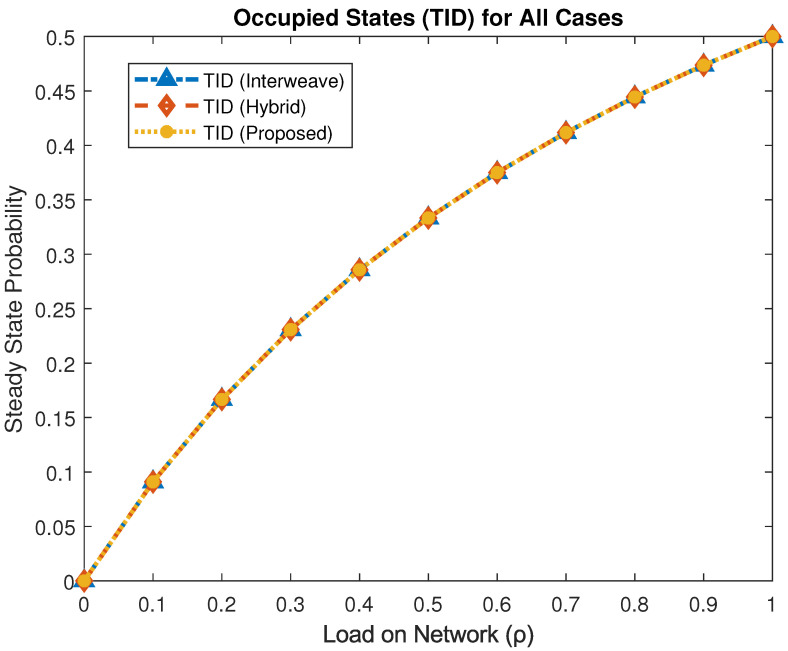
Steady-state probability of TID occupancy under different access schemes.

Figure 5 plots the stationary probability that the TID occupies the channel for the interweave, hybrid, and proposed mechanisms. Across all loads ρ∈[0,1], the TID occupancy remains highest for all schemes, confirming that critical traffic always receives immediate access and QoS guarantees.

**Figure 6:**
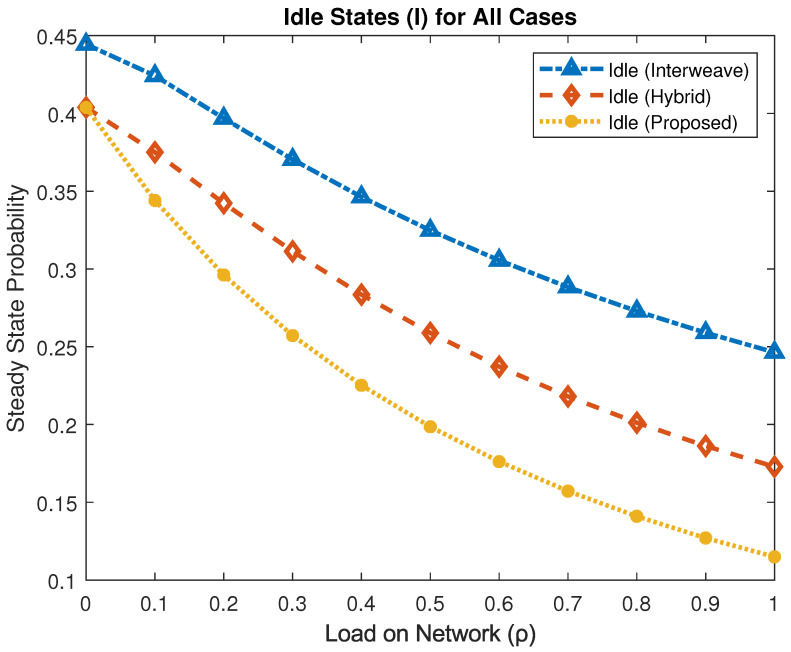
Steady-state probability of the idle state under different access schemes.

Figure 6 compares the idle-state probability, $\pi_{I}$, for the three mechanisms. As the load increases, $\pi_{I}$ decreases, indicating higher spectrum utilization. At ρ=1, the interweave scheme yields $\pi_{I}\approx 0.246$, the hybrid scheme $\pi_{I}\approx 0.172$, and the proposed framework $\pi_{I}\approx 0.114$. Relative to interweave, the proposed framework reduces idle time by approximately 72%, and by 34% compared to hybrid, thereby maximizing spectrum usage.

**Figure 7:**
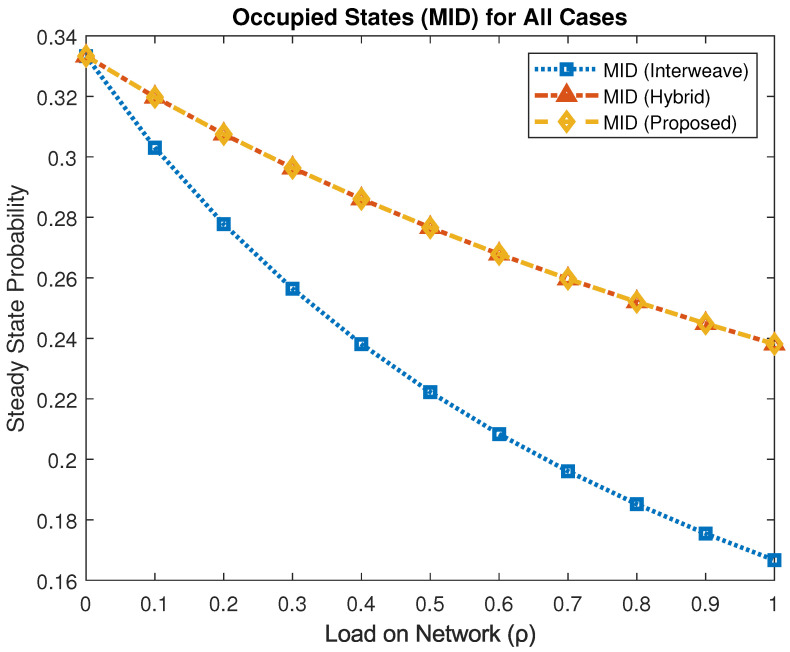
Steady-state probability of MID occupancy under different access schemes.

Figure 7 illustrates the steady-state probability that MID occupies the channel. Under full load (ρ=1), the interweave mechanism yields $\pi_{MID}\approx 0.166$, whereas both the hybrid and the proposed framework increase this to $\pi_{MID}\approx 0.238$. This represents a roughly 35% improvement in MID spectrum access, demonstrating that adaptive coexistence and waiting mechanisms effectively boost utilization for medium-priority traffic.

**Figure 8:**
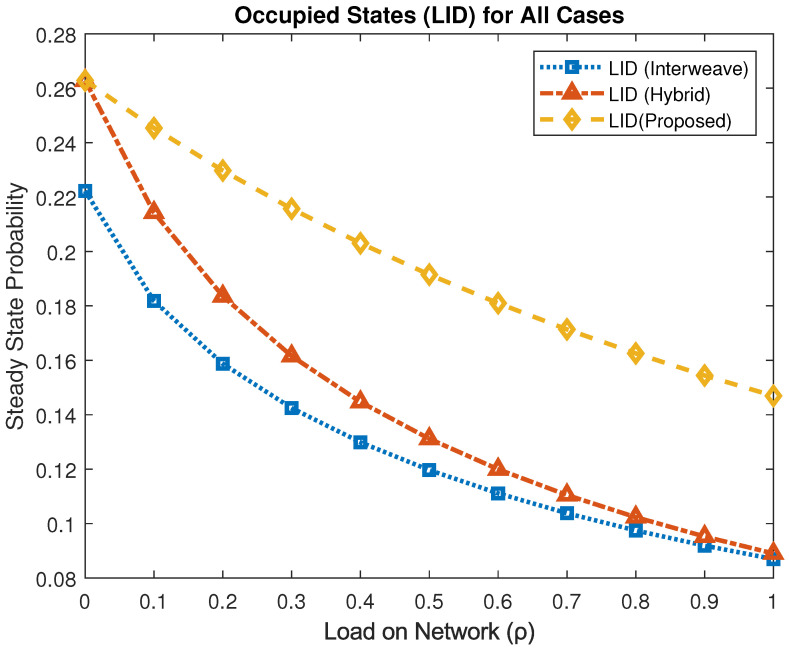
Steady-state probability of LID occupancy under different access schemes.

Figure 8 compares the stationary probability of LID accessing the channel. At ρ=1, the interweave and hybrid schemes achieve $\pi_{LID}\approx 0.086$ and $\pi_{LID}\approx 0.089$, respectively, while the proposed mechanism raises this to $\pi_{LID}\approx 0.146$. This corresponds to improvements of approximately 51% over interweave and 49% over hybrid access, highlighting the effectiveness of the waiting state and handoff strategy in sustaining LID connectivity.

Table 2 summarizes the key advantages and limitations of each spectrum access mode. Interweave access offers interference-free, high-throughput operation but suffers from elevated blocking rates. Underlay access reduces blocking through concurrent transmissions at the expense of energy efficiency due to stringent power caps. The proposed framework unifies these benefits by maximizing utilization and minimizing interruptions, albeit with increased algorithmic complexity and state management overhead.

#### 3.2.5. Computational Complexity Analysis

The computational overhead of the proposed spectrum access framework can be characterized by two primary components. First, the steady-state probability computation, which relies on solving the Continuous-Time Markov Chain model, incurs a complexity on the order of $O(N_{v}^{2})$, where $N_{v}$ denotes the number of vehicles (or CTMC states). Although the introduction of the priority-aware queue and waiting mechanisms increases the number of states and transition rates, this additional complexity is justified by the substantial gains in resource utilization and QoS performance. Second, the real-time spectrum allocation decisions—consisting of priority comparisons and mode selection among interweave, underlay, and coexistence access—are executed in constant time, O(1). These constant-time checks enable immediate reallocation of spectrum resources, ensuring that top- and medium-priority devices experience minimal latency, while the waiting mechanism for low-priority devices effectively reduces session drops without incurring significant computational delay. Together, these two components strike a balance between analytical rigor and operational efficiency, making the framework suitable for deployment in high-mobility vehicular environments.

## 4. Simulation and Results

In this section, a comprehensive evaluation of the proposed spectrum access framework is presented through numerical simulations. We compare its performance against two baseline schemes—pure interweave access and hybrid interweave–underlay access—across seven key QoS metrics: steady-state occupancy, blocking probability, interruption probability, energy efficiency, throughput, spectrum utilization, and EDDT.

The simulation models a highly dynamic V-IoT network with three priority levels. A CTMC captures the state transitions under varying traffic loads, while system parameters are chosen to reflect realistic V-IoT deployments. Table 3 lists the main configuration settings used in our experiments.

All simulation results presented in this work are derived analytically by solving the steady-state equations of the CTMC model. Unlike stochastic or event-driven simulations, this approach yields closed-form expressions for each system state, enabling deterministic evaluation of performance metrics. This method ensures precise, repeatable outcomes without requiring multiple random runs, confidence intervals, or variance computations. The analysis captures long-term average behavior under defined traffic conditions and highlights the QoS trade-offs for different spectrum access mechanisms.

Each simulation run spans the full range of network load (ρ), and metrics are averaged over sufficiently long intervals to ensure convergence of the Markov Chain. In the subsections that follow, we analyze each performance metric in turn, highlighting the relative gains of the proposed framework.

### 4.1. Spectrum Utilization

**Figure 9:**
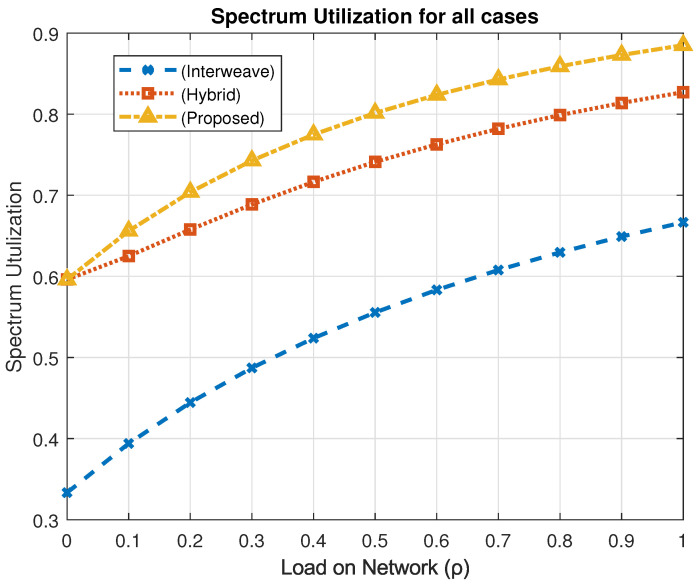
Spectrum utilization across access schemes.

Spectrum utilization, denoted as *U*, represents the proportion of time that the channel is actively used by any device in the network. It is a key performance metric in V-IoT systems, as efficient utilization of scarce spectrum resources directly affects network capacity and QoS delivery. The metric is defined as

(14) $$U=1-\pi_{I},$$

where $\pi_{I}$ is the steady-state probability that the channel remains idle (i.e., not used by any V-IoT user). Figure 9 illustrates the variation of spectrum utilization with respect to the network traffic load (ρ) under the three considered spectrum access schemes—interweave, hybrid, and the proposed PASM framework.

At low traffic loads, all schemes show similar utilization levels, as the channel remains underutilized due to fewer active devices. However, as the traffic load increases, notable differences emerge. Under peak load conditions (ρ=1), the interweave approach achieves a utilization of 66.6%, constrained by its strict reliance on idle channels and lack of concurrent access support. The hybrid access scheme improves utilization to 82.7% by allowing simultaneous access under controlled interference conditions. The proposed PASM framework outperforms both, reaching a spectrum utilization of 88.5%.

This 32.6% improvement over the interweave model and 6.6% gain over hybrid access reflects the effectiveness of our design in minimizing idle channel periods and better leveraging spectrum availability. The coexistence mode in PASM plays a key role by introducing a waiting mechanism for LID users, thereby reducing unnecessary session drops and avoiding idle periods caused by rigid access policies. Moreover, the dynamic switching among interweave, underlay, and coexistence modes enables adaptive allocation aligned with real-time network demand.

Higher spectrum utilization not only implies better bandwidth efficiency but also translates to improved data delivery, reduced blocking, and enhanced support for QoS differentiation across user priority classes. Thus, the proposed framework achieves a more balanced and efficient use of the spectrum, making it particularly suitable for highly dynamic and heterogeneous vehicular communication environments.

### 4.2. Throughput Analysis

Throughput, *T*, quantifies the aggregate data rate delivered by all users and is defined as

$$T=\sum_{\beta }\pi \left(\beta \right)\;R_{\beta },$$

where $R_{\beta }$ is the data rate in state β and π(β) its steady-state probability.

**Figure 10:**
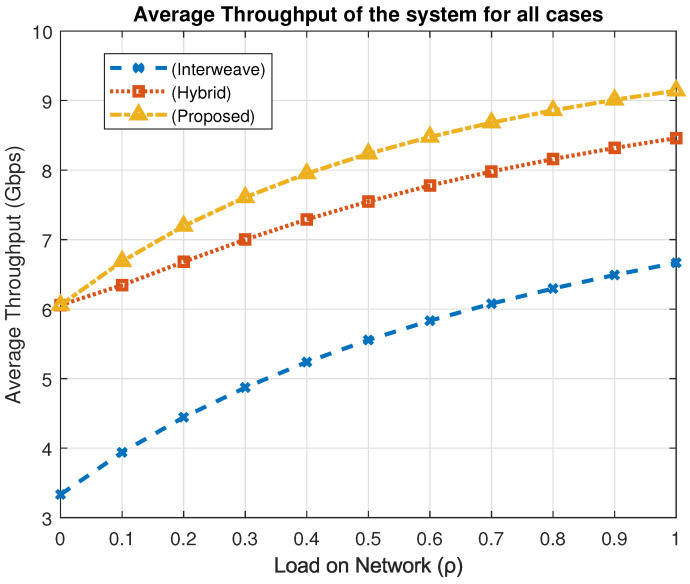
Average throughput across spectrum access schemes.

Figure 10 plots the average throughput versus network load for the three access schemes. Under full load (ρ=1), the interweave approach delivers 6.66 Gbps, the hybrid scheme 8.46 Gbps, and the proposed framework 9.14 Gbps. This corresponds to a 37.2% increase over interweave and an 8.1% gain over hybrid access. The results confirm that by dynamically balancing priority-aware access and coexistence modes, the proposed mechanism maximizes data delivery rates while maintaining low latency for high-priority traffic.

**Figure 11:**
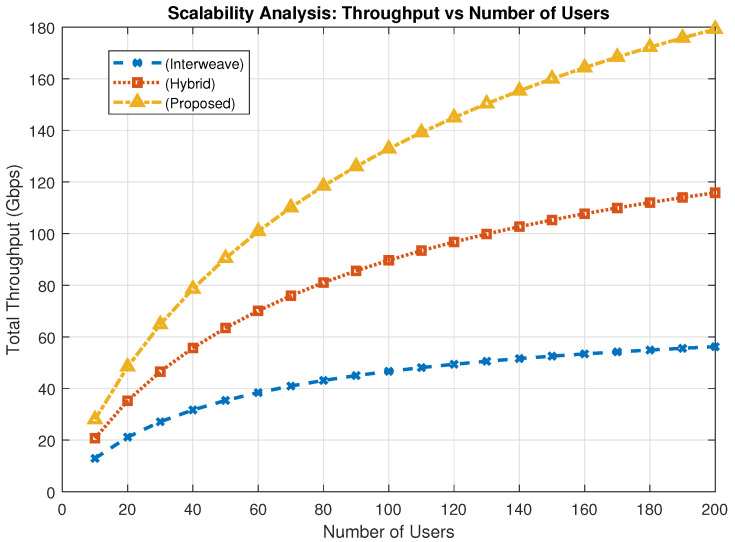
Throughput scalability analysis.

Figure 11 presents the scalability analysis of the proposed PASM framework in terms of average throughput under increasing user density. As the number of users grows, the throughput of all schemes initially increases but begins to saturate at higher densities due to contention and resource constraints. The proposed PASM framework consistently outperforms the interweave and hybrid schemes across all scales, demonstrating better adaptability and resource coordination. This confirms the framework’s robustness and scalability for deployment in high-density vehicular IoT environments.

### 4.3. Blocking Probability Analysis

Blocking probability, $P_{b}$, quantifies the chance that a spectrum access request is denied due to full channel occupancy. It is given by

$$P_{b}=\pi_{t}+\pi_{tm}+\pi_{tl},$$

where $\pi_{t}$, $\pi_{tm}$, and $\pi_{tl}$ represent the steady-state probabilities of denial for TID, TID + MID, and TID + LID combinations, respectively.

The proposed framework mitigates blocking by implementing a buffering strategy for lower-priority LID users, thus enhancing fairness and minimizing service denials across the system.

**Figure 12:**
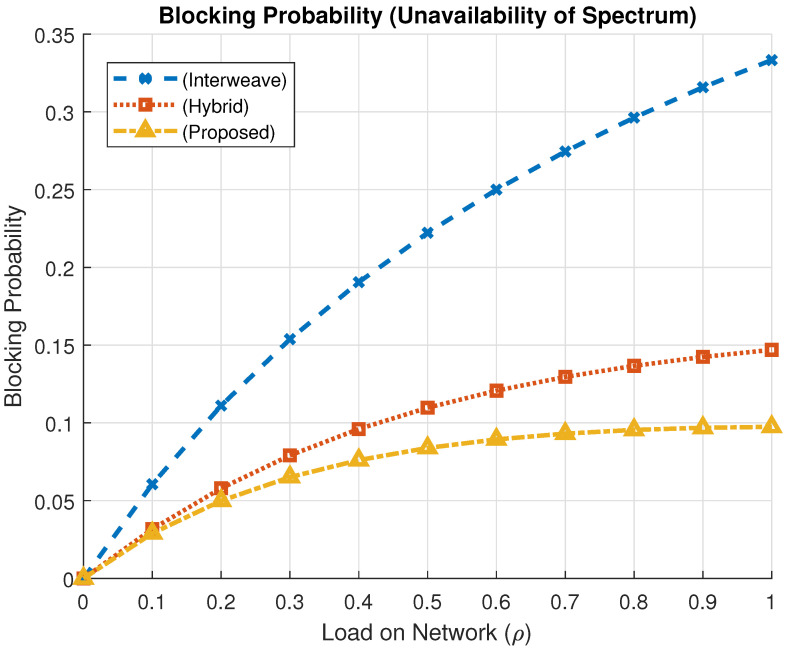
Blocking probability comparison for the case of TID only users.

Figure 12 illustrates the blocking probability in a scenario where only TID users are considered. At maximum traffic load (ρ=1), the interweave scheme yields a blocking probability of 0.3333, the hybrid scheme reduces it to 0.1469, while the proposed mechanism achieves the lowest value of 0.0974. This reflects a reduction of approximately 70.8% compared to interweave and 33.7% relative to the hybrid model.

**Figure 13:**
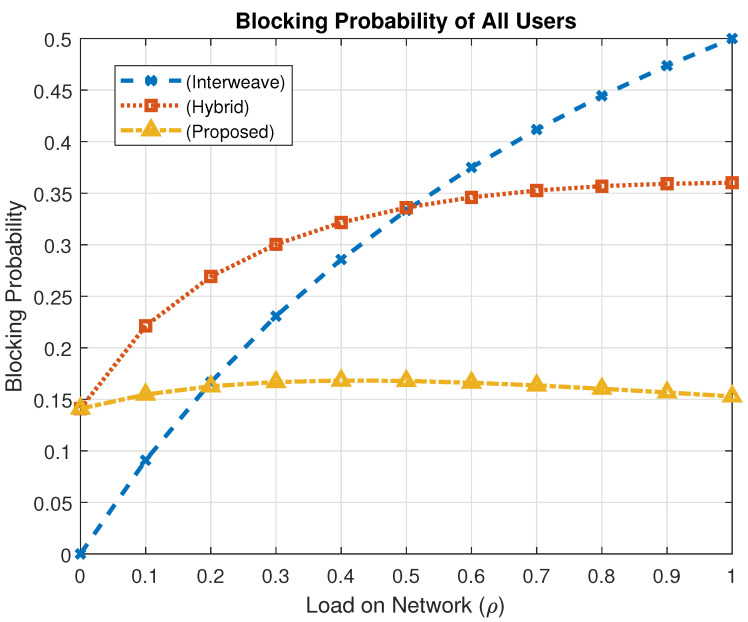
Blocking probability comparison (TID and MID).

Figure 13 presents results when both TID and MID users are active. At full load, the interweave model exhibits a blocking probability of 0.5, the hybrid model achieves 0.3601, and the proposed approach further reduces it to 0.1528. These results indicate a 69.4% reduction over interweave and a 57.6% improvement over the hybrid method, showcasing the efficiency of the proposed spectrum access policy in high-load conditions.

### 4.4. Interruption Probability Analysis

The interruption probability ($P_{int}$) represents the likelihood that an ongoing communication session is prematurely terminated due to preemption by higher-priority users, potentially resulting in degraded service continuity.

**Figure 14:**
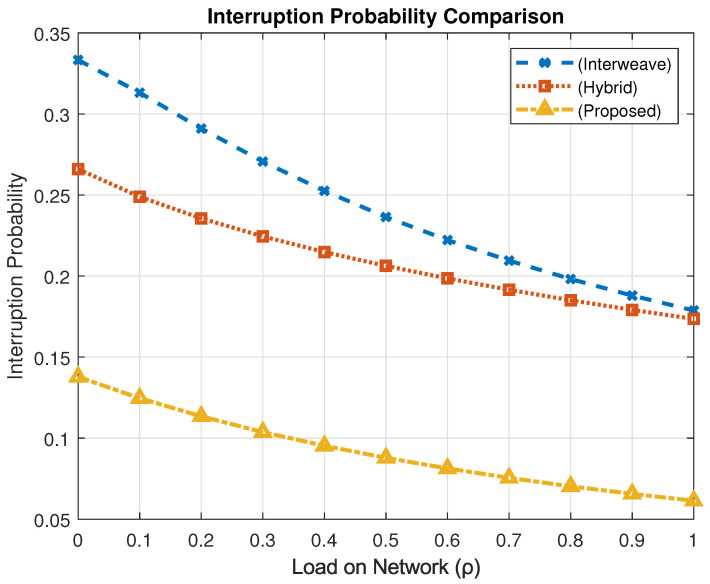
Interruption probability comparison.

Figure 14 compares the interruption probabilities under different spectrum access schemes. At peak traffic load (ρ=1), the interweave model yields an interruption probability of 0.1787, while the hybrid model slightly improves it to 0.1736. The proposed spectrum access framework significantly outperforms both, achieving an interruption probability of only 0.0614—representing an approximate reduction of 65% compared to both baseline models.

These improvements stem from the intelligent design of the proposed mechanism, which incorporates optimized access transition policies and a preemptive queuing strategy. This ensures enhanced session continuity and improved service reliability for medium- and low-priority users without compromising the performance for higher-priority traffic.

### 4.5. Energy Efficiency Analysis

Energy efficiency ($E_{eff}$) is the ratio of successfully transmitted bits to total energy consumed. It is a key performance metric for sustainable vehicular networks. It is calculated as

(15) $$E_{eff}=\frac{\sum R_{x}P_{x}}{\sum P_{\mathrm{total}}},$$

where $R_{x}$ denotes the data rate under access mode *x*, $P_{x}$ is the power consumption in that mode, and $P_{\mathrm{total}}$ represents the total energy expenditure across all modes.

**Figure 15:**
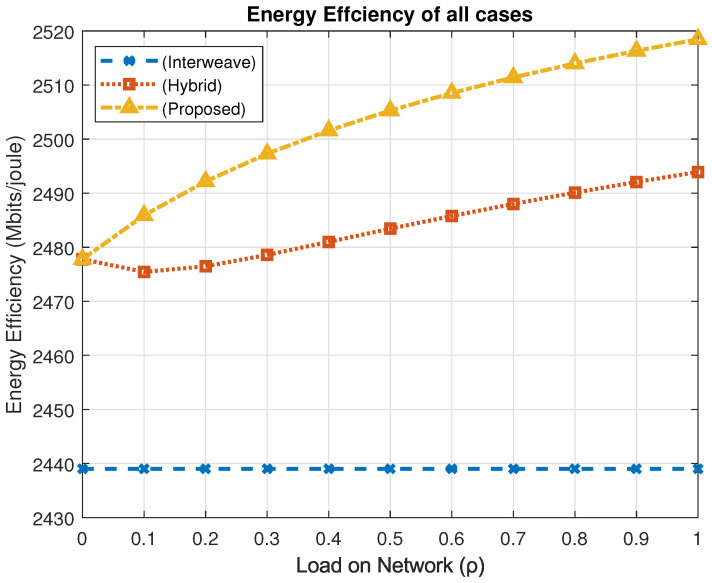
Energy efficiency comparison.

Figure 15 illustrates the comparative energy efficiency of the evaluated spectrum access schemes. At peak traffic load (ρ=1), the interweave model achieves 2.43902 Gbits/joule, the hybrid model improves to 2.49392 Gbits/joule, and the proposed mechanism reaches 2.51846 Gbits/joule.

This translates to an energy efficiency improvement of approximately 3.2% over the interweave model and 1.0% over the hybrid model. These gains are primarily attributed to the proposed framework’s adaptive power control and dynamic spectrum access strategies, which enable energy-aware transmission while maintaining high throughput and service quality.

### 4.6. Extended Data Delivery Time

The *EDDT* measures the average delay experienced during successful data transmission, particularly reflecting the waiting time of low-priority users. It is formulated as

(16) $$\mathrm{EDDT}=\frac{\sum \pi_{LID}\;W_{LID}}{\sum \pi_{LID}},$$

where $W_{LID}$ denotes the waiting time of low-priority (LID) users and $\pi_{LID}$ represents their steady-state occupancy.

**Figure 16:**
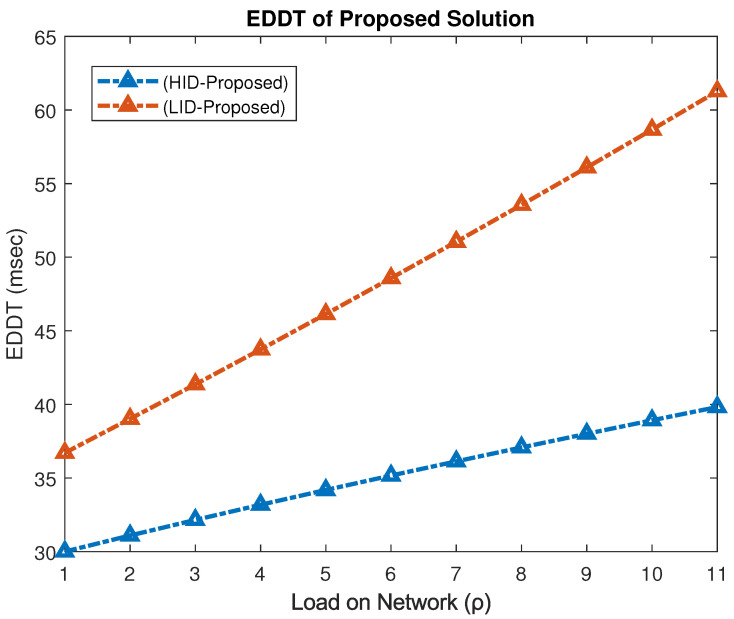
EDDT performance for TID and LID users.

Figure 16 illustrates the EDDT for both TID and LID users under the proposed spectrum access model. At peak load (ρ=1), the proposed scheme achieves an EDDT of 39.82 ms for TID and 61.27 ms for LID users, indicating effective delay management even under high traffic conditions.

**Figure 17:**
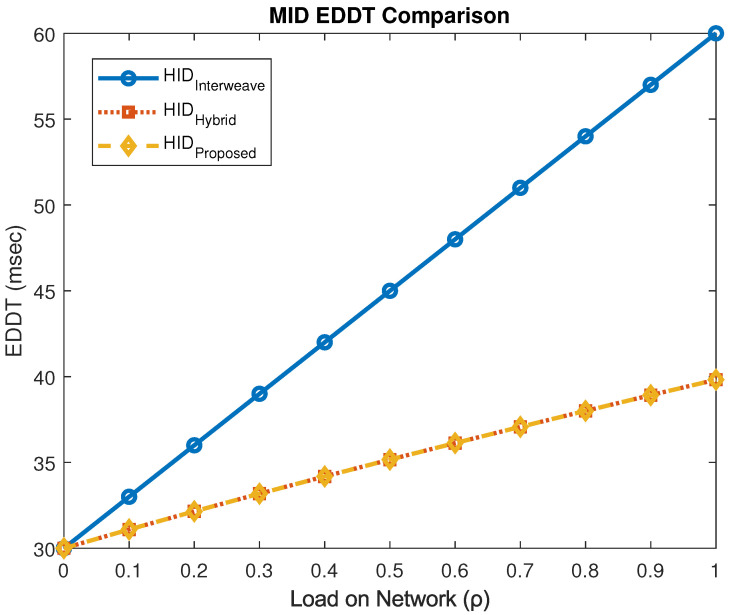
EDDT performance for MID users.

Figure 17 compares the EDDT for MID users across different models. The proposed mechanism significantly reduces the delay to 39.82 ms compared to 60 ms in the interweave model, yielding an improvement of approximately 40%.

**Figure 18:**
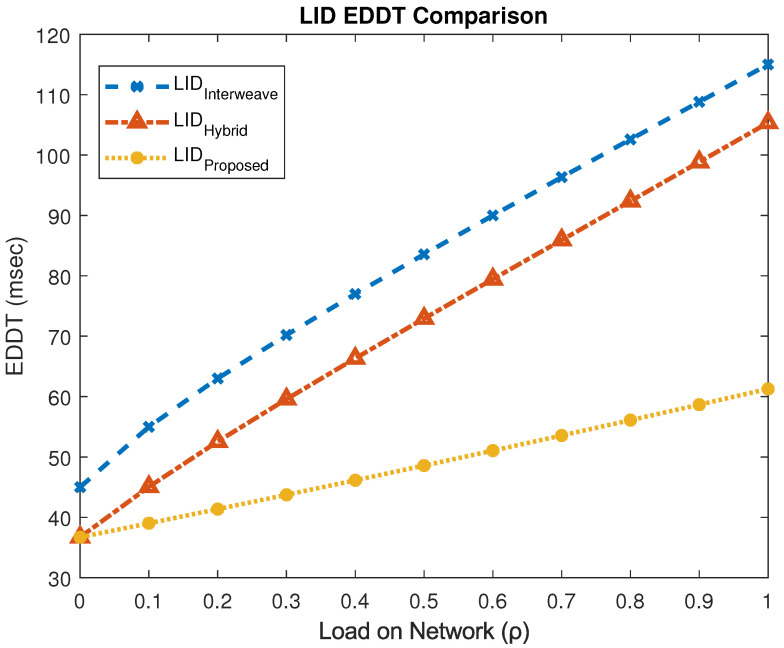
EDDT performance for LID users.

Figure 18 focuses on the EDDT experienced by LID users. The interweave model exhibits the highest delay at 115 ms, while the hybrid and proposed schemes achieve 53.48 ms and 61.27 ms, respectively. This corresponds to a 61% reduction in EDDT compared to the interweave model, albeit with a slight 14% increase relative to the hybrid model.

The proposed framework significantly reduces delivery delays for high- and medium-priority users and substantially outperforms the interweave scheme for low-priority users, thereby enhancing overall system responsiveness and fairness across traffic classes.

Energy–performance trade-off discussion: While the proposed PASM framework demonstrates improved energy efficiency, it is essential to understand its interplay with other performance metrics. As observed in Figure 10, Figure 15 and Figure 18, the system maintains high throughput and fairness, even as energy-saving mechanisms are employed. This balance is achieved by intelligent switching between spectrum access modes, allowing reduced energy usage without significant compromise on service continuity or data delivery rates. However, a marginal increase in delay for low-priority traffic (LID) is noted under high loads, reflecting the inherent trade-off between minimizing energy consumption and sustaining low latency. Such adaptive behavior reinforces PASM’s practical viability in balancing sustainability and performance.

### 4.7. Computational Complexity Analysis

To assess whether managing multiple priority levels and dynamically switching among interweave, underlay, and coexistence modes introduces any bottleneck, we analyze the computational complexity of the proposed PASM framework.

The steady-state probability computation based on the CTMC model results in a state complexity of $O(N_{v}^{2})$, where $N_{v}$ denotes the number of vehicles. Although solving the balance equations involves matrix operations with a worst-case complexity of $O(S^{3})$, where *S* is the number of CTMC states, efficient iterative solvers can reduce this to $O(S^{2})$, making the solution computationally practical.

The real-time decision-making operations, checking channel state, determining device priority, and selecting the spectrum access mode, are rule-based and performed in constant time, i.e., O(1). This ensures that the PASM framework remains scalable and responsive, even in high-density V-IoT environments, without introducing significant overhead.

### 4.8. Comparative Performance Evaluation

To comprehensively assess the effectiveness of the proposed spectrum access framework, its performance is benchmarked against traditional interweave and hybrid access mechanisms across several critical metrics. Table 4 summarizes these results, highlighting the relative improvements offered by the proposed solution.

The results clearly demonstrate that the proposed framework outperforms the interweave and hybrid models across all evaluated dimensions. Notably, it achieves a throughput of 9.14 Gbps under peak load conditions (ρ=1), corresponding to an 8.1% improvement over the hybrid model and a 37.2% increase over the interweave model. Similarly, spectrum utilization reaches 0.885, surpassing the hybrid (0.827) and interweave (0.666) baselines.

By incorporating adaptive, priority-aware spectrum management and preemptive queuing strategies, the proposed mechanism significantly reduces blocking and interruption probabilities while optimizing energy efficiency. The considerable reduction in EDDT for low-priority users enhances overall QoS fairness without compromising the delay performance for high-priority traffic. These findings highlight the proposed framework’s potential for deployment in future V-IoT environments, particularly within 6G-enabled networks, where scalability, energy efficiency, and ultra-reliable low-latency communication are paramount. Its robust adaptability to dynamic traffic demands and heterogeneous priority levels positions it as a strong candidate for next-generation spectrum access solutions.

While the proposed PASM framework is evaluated using a CTMC-based numerical simulation model, which offers analytical tractability and steady-state analysis, it does not fully capture spatial dynamics, real-time vehicular mobility, or practical channel sensing imperfections. Tools such as NS-3 or physical testbeds provide a more detailed view of protocol-level behavior, propagation environments, and mobility interactions. Similarly, the assumption of perfect channel sensing—used here to simplify analysis—may not hold in realistic V-IoT environments where interference, fading, and noise introduce sensing errors such as false alarms and missed detections. These limitations may influence key performance metrics, including blocking probability, interruption probability, and throughput. However, our focus in this work is to evaluate the fundamental performance of spectrum access strategies across different priority classes under controlled conditions. As suggested in [27], future research should integrate mobility-aware simulators and incorporate adaptive sensing techniques, such as dynamic threshold-based energy detection or cooperative sensing, to improve robustness and realism in evaluating the PASM framework.

## 5. Conclusions and Future Work

In this work, we propose a novel priority-aware spectrum management scheme for improving QoS provisioning in V-IoT networks. The proposed solution involves key parameters such as blocking probability, interruption probability, and energy efficiency under an adaptive spectrum access paradigm based on CTMC. Unlike conventional static or hybrid spectrum access schemes, the proposed framework dynamically alternates between interweave, underlay, and coexistence modes, applying energy-aware transmission strategies and waiting policies to prevent service interruption to a significant extent. Our research demonstrates significant improvements, including a substantial reduction in blocking probability and interruption probability, enhanced energy efficiency, increased spectrum utilization, and a decrease in EDDT. These improvements verify that the proposed solution outperforms existing spectrum access techniques and offers a scalable and efficient solution for future next-generation 6G-enabled vehicle networks.

In the future, the proposed PASM framework can be further enhanced through several promising research directions. Machine learning techniques, particularly reinforcement learning, may enable real-time and adaptive spectrum allocation under uncertain and dynamic traffic and channel conditions. Federated learning is another potential avenue, offering privacy-preserving and decentralized spectrum management without relying on centralized data aggregation. Additionally, multi-agent reinforcement learning could facilitate cooperative and scalable decision-making for spectrum access in complex autonomous vehicular environments. The framework may also be extended to support MCCV scenarios, where the presence of non-connected vehicles introduces added uncertainty in spectrum demand estimation and coordination. Furthermore, modeling imperfect channel sensing—by incorporating adaptive techniques such as dynamic threshold-based energy detection or cooperative sensing—can help capture more realistic environmental conditions. Moreover, the integration of lightweight security mechanisms, such as blockchain-based access verification or intrusion-aware spectrum sensing, could further enhance the resilience of PASM against spectrum misuse, spoofing, and unauthorized access. This presents an important avenue for future research in securing spectrum access in vehicular environments. To better evaluate the framework’s performance in practical settings, future work may include simulations using real-world vehicular datasets and mobility traces. Simulation platforms such as NS-3, possibly integrated with mobility models from SUMO, can be used to assess the framework under realistic mobility patterns, including high-speed vehicle movement, dynamic topologies, and frequent spectrum handoffs. These efforts will provide deeper insights into the operational behavior and scalability of PASM. Full-scale simulations covering a broader range of performance metrics in large-scale deployments are also a potential future direction. Collectively, these extensions can significantly improve the robustness, adaptability, and real-world applicability of PASM in next-generation 6G-enabled vehicular networks.

## Figures and Tables

**Table 1:** Comparison of selected spectrum access models.

Reference	Contribution	Limitations
[16]	Interweave and hybrid access using CTMC modeling	No energy-efficiency evaluation; limited to WiFi environments
[20]	Resource allocation strategies for agricultural IoT in 5G	No throughput or delay analysis; not focused on V-IoT
[17]	Hybrid access with age-of-information analysis	Ignores energy efficiency and multi-priority user dynamics
[14]	Comparative study of interweave and underlay access	Lacks mobility modeling; no prioritization or queuing
[15]	Energy-efficient underlay spectrum access framework	Not tailored to vehicular settings; omits throughput/delay
[26]	Hybrid spectrum access for cognitive D2D networks	Limited to WiFi; lacks vehicular context and prioritization
[25]	Service capacity and handoff modeling for CRNs	Primary user behavior not addressed; lacks fairness analysis
[19]	Transient analysis of hybrid access schemes	No energy/interruption analysis; WiFi-centric evaluation
[8]	Adaptive spectrum access with multi-mode CTMC for V-IoT	No modeling of energy use or interruption probability
[21]	Federated deep reinforcement learning for decentralized DSA in IoT networks	Assumes stable federated training; limited discussion on vehicular mobility constraints
[22]	Hierarchical adaptive federated RL for resource allocation in IoT networks	No support for priority-aware queuing; not specialized for vehicular mobility scenarios
[23]	Multi-agent D3QN for dynamic resource allocation in vehicular networks	No modeling of priority differentiation or preemptive queuing in multi-class V-IoT
[24]	Blockchain-based secure spectrum sensing and auction for IoT	Lacks support for real-time mobility and multi-priority access in V-IoT

**Table 2:** Advantages and limitations of spectrum access modes.

Mode	Advantages	Limitations
Interweave	Interference-free, highest throughput	High blocking probability under load
Underlay	Low blocking probability, continuous access	Reduced energy efficiency due to power constraints
Proposed	Maximized utilization, fewer interruptions	Increased system complexity and state management

**Table 3:** Simulation parameters.

Parameter	Value
Number of channels	1
Traffic load, ρ	0.0–1.0
TID arrival rate, $\lambda_{t}$	4 requests/s
TID departure rate, $\mu_{t}$	4 requests/s
MID arrival rate, $\lambda_{m}$	1 request/s
MID departure rate, $\mu_{m}$	2 requests/s
LID arrival rate, $\lambda_{l}$	1 request/s
LID departure rate, $\mu_{l}$	2 requests/s
Full-rate transmission, $R_{i}$	10 Gbps
Reduced-rate transmission, $R_{u}$	500 Mbps
Channel bandwidth, *W*	500 MHz
Average file size	100 MB

**Table 4:** Performance comparison of spectrum access mechanisms. The arrows represent the direction of change in value.

Metric	Interweave	Hybrid	Proposed	Improvement (%)
Spectrum utilization	0.666	0.827	0.885	↑ 32.9%
Blocking probability	High	Moderate	Low	↓ 78.6%
Interruption probability	0.1787	0.1736	0.0614	↓ 65.6%
Energy efficiency (Gbits/Joule)	2.43902	2.49392	2.51846	↑ 3.2%
Throughput (Gbps)	6.66	8.46	9.14	↑ 37.2%
EDDT (LID users, ms)	115.00	53.48	61.27	↓ 46.7%

## Data Availability

Dataset available on request from the authors.
